# The Madagascar palm genome provides new insights on the evolution of Apocynaceae specialized metabolism

**DOI:** 10.1016/j.heliyon.2024.e28078

**Published:** 2024-03-14

**Authors:** Clément Cuello, Hans J. Jansen, Cécile Abdallah, Duchesse-Lacours Zamar Mbadinga, Caroline Birer Williams, Mickael Durand, Audrey Oudin, Nicolas Papon, Nathalie Giglioli-Guivarc'h, Ron P. Dirks, Michael Krogh Jensen, Sarah Ellen O'Connor, Sébastien Besseau, Vincent Courdavault

**Affiliations:** a Biomolécules et Biotechnologies Végétales, EA2106, Université de Tours, 37200, Tours, France; b Future Genomics Technologies, 2333 BE, Leiden, the Netherlands; c Univ Angers, Univ Brest, IRF, SFR ICAT, F-49000, Angers, France; d Novo Nordisk Foundation Center for Biosustainability, Technical University of Denmark, Kgs, Lyngby, Denmark; e Department of Natural Product Biosynthesis, Max Planck Institute for Chemical Ecology, Jena, 07745, Germany

**Keywords:** *Apocynaceae*, Evolution, Biosynthetic gene clusters, Specialized metabolites, Alkaloids

## Abstract

Specialized metabolites possess diverse interesting biological activities and some cardenolides- and monoterpene indole alkaloids- (MIAs) derived pharmaceuticals are currently used to treat human diseases such as cancers or hypertension. While these two families of biocompounds are produced by specific subfamilies of *Apocynaceae*, one member of this medicinal plant family, the succulent tree *Pachypodium lamerei* Drake (also known as Madagascar palm), does not produce such specialized metabolites. To explore the evolutionary paths that have led to the emergence and loss of cardenolide and MIA biosynthesis in *Apocynaceae*, we sequenced and assembled the *P. lamerei* genome by combining Oxford Nanopore Technologies long-reads and Illumina short-reads. Phylogenomics revealed that, among the *Apocynaceae* whose genomes have been sequenced, the Madagascar palm is so far the species closest to the common ancestor between MIA producers/non-MIA producers. Transposable elements, constituting 72.48% of the genome, emerge as potential key players in shaping genomic architecture and influencing specialized metabolic pathways. The absence of crucial MIA biosynthetic genes such as strictosidine synthase in *P. lamerei* and non-*Rauvolfioideae* species hints at a transposon-mediated mechanism behind gene loss. Phylogenetic analysis not only showcases the evolutionary divergence of specialized metabolite biosynthesis within *Apocynaceae* but also underscores the role of transposable elements in this intricate process. Moreover, we shed light on the low conservation of enzymes involved in the final stages of MIA biosynthesis in the distinct MIA-producing plant families, inferring independent gains of these specialized enzymes along the evolution of these medicinal plant clades. Overall, this study marks a leap forward in understanding the genomic dynamics underpinning the evolution of specialized metabolites biosynthesis in the *Apocynaceae* family, with transposons emerging as potential architects of genomics restructuring and gene loss.

## Abbreviations

ADH: alcohol dehydrogenase
BUSCO: Benchmarking Universal Single-Copy Orthologs
GO: geissoschizine oxidase
Ks: synonymous substitutions per synonymous sites
LAMT: loganic acid *O*-methyltransferase
LTR: long terminal repeat
MEP: methylerythritol phosphate
MIA: monoterpene indole alkaloid
P450: cytochrome P450
PAS: precondylocarpine acetate synthase
SAT: stemmadenine-*O*-acetyltransferase
SGD: strictosidine β-D-glucosidase
SLS: secologanin synthase
STR: strictosidine synthase
TDC: tryptophan decarboxylase
TE: transposable element
WGD: whole genome duplication

## 1 Introduction

Comprising around 350 genera and 5000 species, the *Apocynaceae* family, commonly known as the dogbane family, exhibits a global distribution, thriving in varied ecosystems from tropical rainforests to arid deserts [1]. Plants from this family showcase a plethora of morphological structures, reproductive strategies, physiological adaptations and phytochemical diversity that have evolved over millennia [[2], [3], [4], [5]]. In addition to its ecological significance, *Apocynaceae* has been a source of invaluable contributions to human health and well-being through its rich specialized metabolites repertoire including monoterpene indole alkaloids (MIAs) and cardenolides [6,7].

Cardenolides are a group of biocompounds produced by plant species mainly belonging to the *Apocynaceae*, *Plantaginaceae*, and *Brassicaceae* families [7,8]. They exhibit a broad range of biological activities including cardiotonic and anti-tumor activities [9,10]. They are characterized by a steroid core structure and a lactone ring [11]. The biosynthesis of cardenolides starts with the condensation of two farnesyl-diphosphates by squalene synthase [12]. From squalene, several enzymatic routes are proposed to generate the three putative sterol precursors of cardenolides: cholesterol, β-sitosterol and campesterol [[13], [14], [15]]. These three precursors can be used as substrates by DpCYP87A106 and CpCYP87A103 to produce pregnenolone [16]. In *Digitalis purpurea*, pregnenolone is further modified by 3β-hydroxysteroid dehydrogenase and progesterone 5β-reductase to produce pregnanolone [[17], [18], [19], [20]]. Even though extensive research has been performed on sterol-derived cardenolides biosynthesis, enzymatic steps from pregnanolone towards the different types of cardenolides remains unidentified in any plant species.

**Fig. 1:**
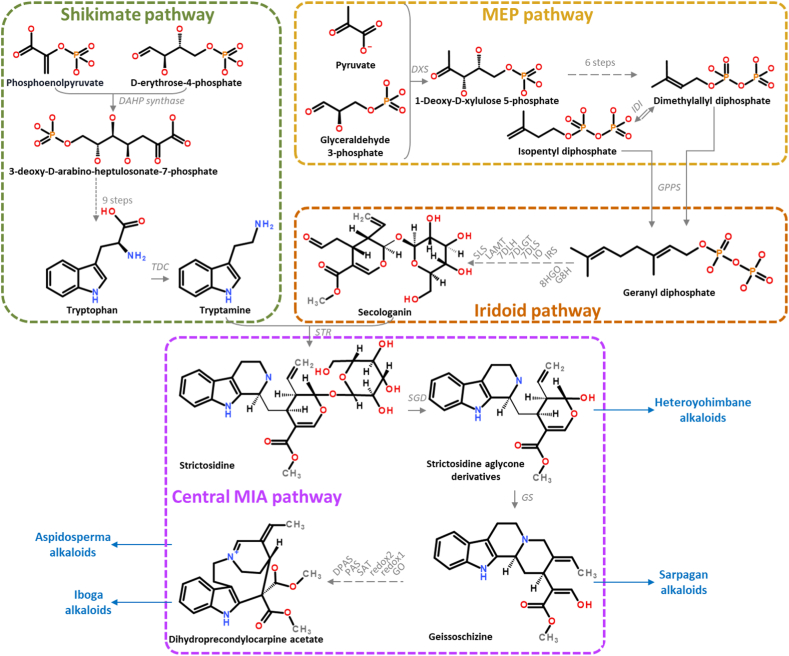
**Schematic representation of monoterpene indole alkaloids (MIA) biosynthetic pathway.** MEP: methylerythritol phosphate, DAHP: 3-deoxy-D-arabino-heptulosonate-7-phosphate, TDC: tryptophan decarboxylase, IDI: isopentenyl-diphosphate Delta-isomerase, DXS: 1-deoxy-D-xylulose 5-phosphate synthase, GPPS: geranyl-pyrophosphate synthase, SLS: secologanin synthase, LAMT: loganic acid *O*-methyltransferase, 7DLH: 7-deoxyloganic acid hydroxylase, 7DLGT: 7-deoxyloganetic acid glucosyl transferase, 7DLS: 7-deoxyloganetic acid synthase, IO: iridoid oxidase, IRS: iridoid synthase, 8HGO: 8-hydroxygeraniol oxidoreductase, G8H: geraniol 8-hydroxylase, STR: strictosidine synthase, SGD: strictosidine β-D-glucosidase, GS: geissoschizine synthase, GO: geissoschizine oxidase, SAT: stemmadenine *O*-acetyltransferase, PAS: precondylocarpine acetate synthase, DPAS: dihydroprecondylocarpine acetate synthase.

MIAs are a group of specialized metabolites produced by plant species mainly belonging to the *Apocynaceae*, *Loganiaceae* and *Rubiaceae* families (*Gentianales* order) and a few *Nyssaceae* (*Cornales* order) [6]. In the *Apocynaceae* family, MIAs are specifically produced by the *Rauvolfioideae* subfamily [2]. Non-MIA producing *Apocynaceae* species are thus reported as non-*Rauvolfioideae* species. A broad range of MIAs exhibit a wide range of biological activities, including anti-tumor, anti-inflammatory, and anti-microbial properties [6,21]. MIA backbones are characterized by an indole ring and a monoterpene unit, which are biosynthetically derived from the shikimate and monoterpene iridoid pathways, respectively [22]. The shikimate pathway starts with the condensation of phosphoenolpyruvate and D-erythrose-4-phosphate by the 3-deoxy-D-arabino-heptulosonate-7-phosphate synthase followed by nine steps resulting in the formation of tryptophan (Fig. 1) [23]. This essential amino acid is further decarboxylated by tryptophan decarboxylase (*TDC*) forming tryptamine, the indole precursor of MIA [24]. The monoterpene iridoid moiety is synthesized from geranyl diphosphate produced by geranyl-diphosphate synthase from isopentyl-diphosphate and from dimethylallyl-diphosphate synthesized from the MEP pathway (Fig. 1) [25]. A series of seven enzymes leads to the synthesis of loganic acid from geranyl-pyrophosphate [26]. Loganic acid is further *O*-methylated by loganic acid O-methyltransferase (*LAMT*) and then converted to secologanin, the monoterpene iridoid precursor of MIA, by secologanin synthase (*SLS*) (Irmler et al., 2000; Murata et al., 2008; Dugé de Bernonville et al., 2015) (Fig. 1) [[27], [28], [29]]. Over the last three decades, the biosynthesis of MIAs has been extensively studied in the iconic plant *Catharanthus roseus* also known as the Madagascar periwinkle [30]. The pathway starts with the condensation of tryptamine and secologanin by strictosidine synthase (*STR*), the first identified Pictet Spengler enzyme [31,32]. The resulting intermediate and first MIA of the whole pathway, strictosidine, is then deglycosylated by strictosidine β-D-glucosidase (*SGD*) [33,34] and further modified by a series of enzymatic reactions to produce dihydroprecondylocarpine acetate, the last molecule that can be considered as part of a central MIA pathway (Fig. 1) [35]. This central MIA pathway contains several branching points resulting to the wide range of MIAs (Fig. 1). Strictosidine aglycones are the precursors of heteroyohimbane and yohimbane alkaloids such as the antihypertensive ajmalicine [[36], [37], [38], [39], [40]]. Geissoschizine is the precursor of sarpagane alkaloids such as the antiarrhythmic ajmaline [41,42]. Dihydroprecondylocarpine is the precursor of iboga alkaloids such as the antiaddictive ibogaine [43,44] and aspidosperma alkaloids such as the anticancer precursor vindoline [45,46]. The identification of the MIA biosynthesis molecular actors in several plant species has progressively paved the way for the development of alternative biotechnological processes for MIA-derived pharmaceutical supply, notably by reconstructing the plant biosynthetic pathways in heterologous organisms [[47], [48], [49]]. It has also shed light on evolutionary trends of the biosynthesis of MIA within plant clades, which has probably co-opted genes from the flavonoid metabolism, as illustrated with *O*-methyltransferases in *C. roseus* and *Camptotheca acuminata* [50,51]. In addition, an interesting characteristic of the MIA pathway is that some biosynthetic genes are physically linked in the genomes, forming biosynthetic gene clusters as described in *C. roseus* [[52], [53], [54], [55], [56]], *Vinca minor* [57] and *Gelsemium sempervirens* [53]. Interestingly, STR and TDC, two key enzymes in MIA biosynthesis, are described to form a biosynthetic gene cluster in many species including *C. roseus* [52,53,56,57], *Rhazya stricta* [58] and *G. sempervirens* [53]. However, no study has so far addressed the evolution of the capacity of synthesizing MIAs among plant families and subfamilies.

**Fig. 2:**
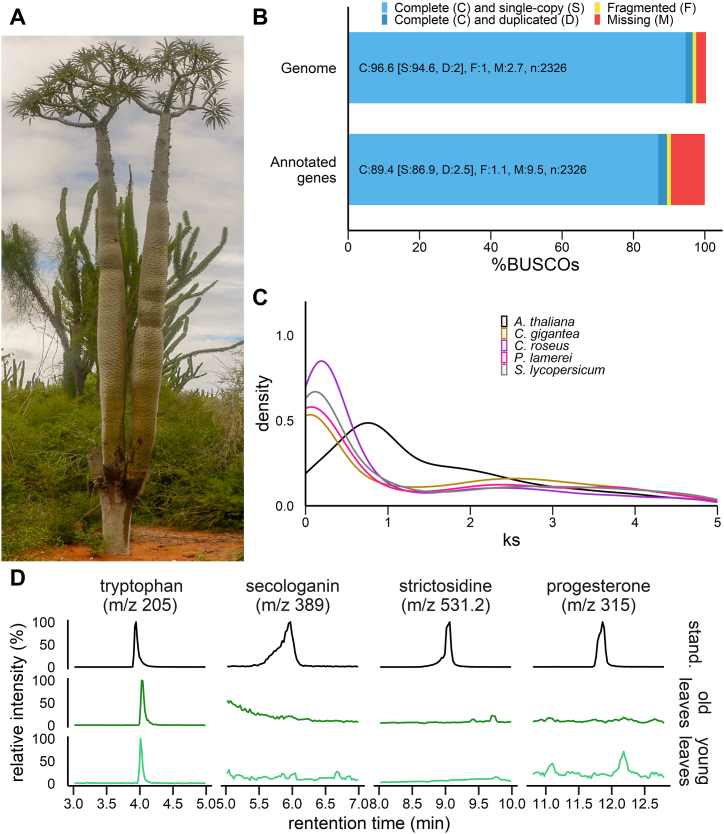
**General characteristics of the assembled genome and specialized metabolite profile from *P. lamerei*.** (A) *P. lamerei*, commonly known as Madagascar palm tree, is a succulent spiky tree. (B) BUSCO scores of genome and annotated genes based on *Eudicotyledons* lineage. (C) Synonymous substitution rate (Ks) distribution plot for *P. lamerei* paralogs compared to other *Eudicotyledons*. (D) Tryptophan (*m*/*z* 205), secologanin (*m*/*z* 389), strictosidine (*m*/*z* 531.2) and progesterone (*m*/*z* 315) profiles of *P. lamerei* old and young leaves.

In this context, *Pachypodium lamerei* Drake, commonly known as the Madagascar palm, stands out as an interesting species. *P. lamerei* is indeed a succulent native to Madagascar, well adapted to arid regions and featuring a massive spiky trunk with thick leaves at the top (Fig. 2A). This stunning tree belongs to the *Apocynoideae* subfamily of *Apocynaceae*. Conversely to plants from the *Rauvolfioideae* subfamily of *Apocynaceae* (*e.g. C. roseus*, *V. minor*, *Voacanga thouarsii* or *Rauvolfia* species) that are well-documented for producing a large palette of MIAs and plants from the *Asclepiadoideae* subfamily (*e.g. Calotropis procera*) that are well-documented for producing cardenolides, *P. lamerei* does not biosynthesize such specialized metabolites (Fig. 2D) thus questioning the evolution processes associated to this incapacity in non-*Rauvolfioideae* and non-*Asclepiadoideae* species [2,5,53,[59], [60], [61]]. In this study, we report the first *Pachypodium* genome assembly. We propose several evolutionary scenarios, notably including transposable elements (TE), for the emergence and loss of specialized metabolites biosynthesis pathways.

## 2 Results and discussion

### 2.1 Genome sequencing, assembly and annotation

Flye (v.2.8.3) was used to assemble the *P. lamerei* genome using Oxford Nanopore Technologies (ONT) long-reads, resulting in a 968.8 Mb assembly spread across 3029 scaffolds. A double polishing using Illumina short-reads and pilon (v.1.23) next resulted in a 968.6 Mb assembly distributed across 3029 scaffolds (Table 1). Interestingly, this genome is the second largest *Apocynaceae* genome available after *V. thouarsii* (1351.2 Mb, [62]). A clear sign of the excellent quality of the assembled genome is the base-level QV of 31.1, which corresponds to more than 99.999% correctness, and the k-mer completeness of 92.5% (Table 1). Overall, the assembled genome is 96.6% complete (Fig. 2B) with a low duplication rate (2.0%), according to the identification of core *Eudicotyledons* Benchmarking Universal Single-Copy Orthologs (BUSCO). This BUSCO score is similar to the other *Apocynaceae* genomes (Supplementary Figure S1A) with the exception of *Apocynum venetum* and *V. minor*, both presenting a slightly higher duplication (14.4% and 36.6%, respectively), and *Calotropis procera*, being moderately less complete (85.3%).

**Table 1:** Genome assembly features of *Pachypodium lamerei*.

Length of genome assembly (bp)	968,622,212
Number of scaffolds	3029
N50 of scaffolds (Mb)	1.67
L50 of scaffolds	145
GC content (%)	35.34
Longest scaffold (Mb)	10.422
scaffolds >50 kb (%)	96.75
QV	31.1
Number of protein-coding genes	16,176
Average gene length (bp)	5434
Average transcripts number per gene	1.6

**Fig. 3:**
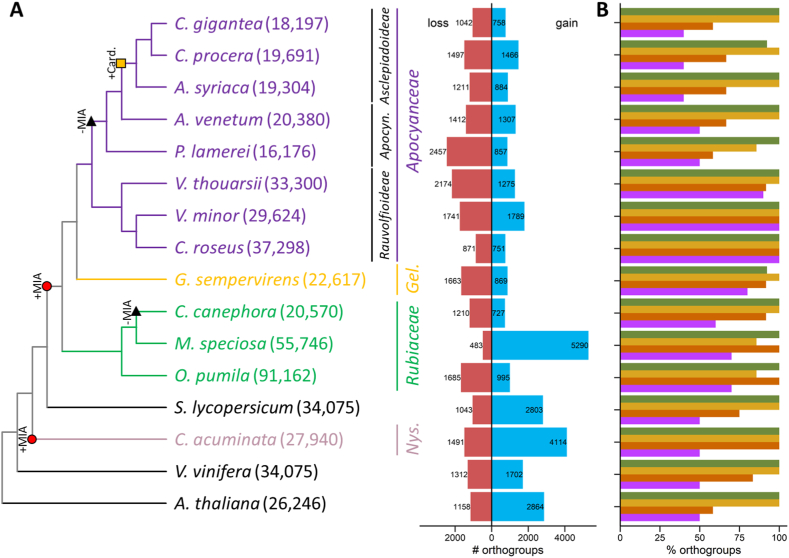
**Comparative genomic analysis of *P. lamerei* and 15 other plant species.** (A) Phylogenetic tree. Colored branches represent MIA-producing plant families including eight *Apocynaceae* (purple), one *Gelsemiaceae* (Gel., yellow), three *Rubiaceae* (green) and one *Nyssaceae* (Nys., pink). Apoc.: *Apocynoideae* subfamily. Species: *C. gigantea: Calotropis gigantea, C. procera: Calotropis procera, A. syriaca: Asclepias syriaca, A. venetum: Apocynum venetum, P. lamerei: P. lamerei, V. thouarsii: Voacanga thouarsii, V. minor: Vinca minor, C. roseus: Catharanthus roseus, G. sempervirens: Gelsemium sempervirens, C. canephora: Coffea canephora, M. speciosa: Mytragyna speciosa, O. pumila: Ophiorriza pumila; S. lycopersicum: Solanum lycopersicum, C. acuminata: Camptotheca acuminata, V. vinifera: Vitis vinifera, A. thaliana: Arabidopsis thaliana*. Numbers in brackets represent the number of annotated genes in each genome. Red circles correspond to MIA biosynthesis appearance events (+MIA). Black triangles correspond to MIA biosynthesis disappearance events (-MIA). Yellow square corresponds to cardenolides biosynthesis appearance events (+card.). For each species, orthogroups gain (blue, right) and loss (red, left) are shown on the barplot. (B) Percent of MIA biosynthesis related orthogroups found in each species. Green: indole, orange: MEP, Brown: Iridoid, Violet: Central MIA pathway. (For interpretation of the references to colour in this figure legend, the reader is referred to the Web version of this article.)

On the basis of available resources, genomes of MIA-producing *Apocynaceae* (*i.e.* from the *Rauvolfioideae* subfamily) generally contain around 30,000 genes (*C. roseus*: 37,298 genes [55]; *V. thouarsii*: 33,300 genes [62]; *V. minor*: 29,624 genes [57]). Despite being the second largest *Apocynaceae* genome characterized to date, *P. lamerei* displays the lowest number of genes (16,176 protein encoding genes) (Fig. 3A). Interestingly, *Gelsemium sempervirens*, a MIA-producing *Gentianales* close to *Apocynaceae* (Fig. 3A), features relatively similar gene contents than non-*Rauvolfioideae* species genomes (*i.e. C. gigantea*, *C. procera*, *Asclepias syriaca*, *A. venetum* and *P. lamerei*) (Fig. 3A). This suggests that the higher gene content in *Rauvolfioideae* species likely results from gene duplication occurring after *Rauvolfioideae*/non-*Rauvolfioideae* divergence. We also noted that all these predicted gene sets achieved good completeness scores according to *Eudicotyledons* BUSCO, ranging from 81% (*C. procera*) to 92.6% (*A. venetum*) (Supplementary Figure S1B). In addition, the combination of BLASTp and BLASTx against UniProt database and hmmscan against the Pfam database led to the functional annotation of 85.2% of the predicted genes (13,777 of the 16,176 genes, Supplementary Table S1).

Upon the identification of paralogous gene pairs in each species and estimation of the synonymous substitutions per synonymous site (Ks) for each gene pair, whole genome duplication (WGD) events were investigated. Paralogs were thus arranged according to age (Ks). In this case, genes being contemporarily duplicated showed a high-density peak at low Ks. Duplicated genes progressively disappear ending in an L-shape pattern. Important increases in paralogs at a particular Ks are caused by large-scale duplication such as WGD events. These events usually appear as secondary peaks in the Ks plot. *Arabidopsis thaliana*, *C. roseus*, *P. lamerei* and *Solanum lycopersicum* all showed a secondary peak at around Ks = 2.5 (Fig. 2C), which corresponds to the well-described and conserved whole-genome triplication event common to *Eudicotyledons* [63]. No additional peak could be identified in *P. lamerei* Ks plot, thus indicating the absence of any other recent WGD event.

Since TE are known to play important roles in genetic instability and genome evolution [64], the TE composition of the *P. lamerei* genome was also inferred. Based on this analysis, it appears that 72.48% of *P. lamerei* genome is composed of TE, most of them being long terminal repeat (LTR, Supplementary Figure S2). The majority of *P. lamerei* LTR are Copia-type (37.41%), Gypsy-type LTR accounting for 17.58% (Supplementary Figure S2). Such Copia-Gypsy ratio is mainly found in *Rauvolfioideae* species while non-*Rauvolfioideae* tend to have a Copia-Gypsy ratio of 1 or less (Supplementary Figure S2). In accordance with their genome size, *P. lamerei* displays the second highest proportion of TE after *V. thouarsii* [62]. The similar genome size and TE proportion in these two species suggest that *P. lamerei* and *V. thouarsii* are likely among the closest species to a common ancestor that led to the differentiation of *Rauvolfioideae* and non-*Rauvolfioideae Apocynaceae* species.

### 2.2 Conservation of specialized metabolism biosynthetic genes

To ensure completeness of the predicted proteome, we first looked for orthologs of genes involved in the biosynthesis of flavonoids, a prominent group of specialized metabolites broadly conserved in flowering plants. We first performed a targeted metabolomics analysis on key intermediates of the flavonoid pathway to ensure their occurrence in the studied species *P. lamerei* and the MIA-biosynthesis model plant *C. roseus*. As previously reported in *C. roseus* [65,66], we mainly identified final products of the flavonoids pathway such as glycosylated flavonols (*i.e.* kaempferol-3-glucoside and quercetin-3-glucoside) and both anthocyanidins and anthocyanins (*i.e* cyanidin and pelargonidin) in both species (Table 2). We also identified the flavonoid precursor phenylalanine. Interestingly, no flavan-3-ols (*i.e.* catechin and epicatechin) could be detected in the analyzed samples. As flavonoids could be detected in *P. lamerei*, we thus performed a BLASTp analysis of functionally characterized proteins from *A. thaliana*, *Vitis vinifera*, *C. roseus* and *Desmodium uncinatum* against the *P. lamerei* and *C. roseus* predicted proteomes (Supplementary Tables S2-S3). We were able to identify high confidence orthologs with similar identity and coverage between the two species for the eight genes involved in the biosynthesis of kaempferol, quercetin, pelargonidin, and cyanidin from the condensation of 4-coumaroyl-CoA and 3-malonyl-CoA (Supplementary Table S4). This is in accordance with the detection of the glycone form of all these end products in *P. lamerei* and *C. roseus*. Conversely, no catechin nor epicatechin was detected in *P. lamerei* and *C. roseus* samples corroborating with our incapacity to identify high confidence orthologs for leucoanthocyanidin reductase and anthocyanidin reductase in both species. These results confirmed the gene annotation quality and that no orthologs would potentially escape our identification procedure.

**Table 2:** Detected flavonoids in *P. lamerei* and *C. roseus*.

Metabolite			*P. lamerei*		*C. roseus*		
	Molar mass	RT^a^	Young leaves	Old leaves	Flowers	Young leaves	Old leaves
phenylalanine	165.19	2.7	++	++	++	++	++
naringenin	272.25	12.5	–	–	–	–	–
kaempferol	286.23	13.0	–	–	–	–	–
kaempferol-3-glucoside	448.38	8.9	+	–	++	+	–
quercetin	302.25	11.3	–	–	–	–	–
quercetin-3-glucoside	464.38	7.8	++	–	++	–	–
pelargonidin	271.24	7.9	–	+	+	++	++
cyanidin	287.24	6.9	+	+	+	–	–
cyanidine-3-glucoside	484.83	5.9	+	+	+	+	+
catechin	290.26	4.8	–	–	–	–	–
epicatechin	290.26	5.9	–	–	–	–	–

a Retention time.

On this basis, we next performed a similar analysis with MIA and cardenolides biosynthetic genes. We created a database containing functionally characterized proteins involved in shikimate, MEP, iridoid, MIA and cardenolides pathways [67]. We then performed a BLASTp analysis of this database against predicted proteomes of all studied species and conserved hits of at least 90% coverage and 50% identity (Supplementary Tables S5-S20). We deliberately used low identity and coverage thresholds to maximize ortholog identification probability. As expected, this resulted in a higher number of putative orthologs for multigenic families such as alcohol dehydrogenases (ADHs) and cytochromes P450 (P450s). For instance, these low thresholds led to the identification of 10 SLS-like genes in *C. roseus* while only four *bona fide* SLS exist in this species (Supplementary Table S21). Based on this approach, we were able to retrieve orthologs from genes of shikimate, MEP, steroids and cardenolide pathways for all species with only few exceptions probably due to phylogenetic distance (Supplementary Table S21-S22). We also identified orthologs for most of the genes of iridoid pathway for all *Apocynaceae* with the exception of 8-hydroxygeraniol oxidoreductase and loganic acid *O*-methyltransferase for all non-*Rauvolfioideae* species (50–90% identity, 94–100% coverage).

Interestingly, concerning the key MIA biosynthetic enzyme *STR*, only MIA-producing *Gentianales* present at least one Cr*STR* orthologous gene while no ortholog was identified in other species. As previously reported, *C. acuminata* (*Cornales*) uses an alternative seco-iridoid pathway producing strictosidinic acids via strictosidinic acid synthase that may explain why no CrSTR-like enzyme could be found in this species [68]. It also suggests that MIA biosynthesis in *Gentianales* and *Cornales* may have emerged independently (*i.e.* by convergent evolution). Similarly, no CrSTR-like enzyme has been identified in *Apocynoideae* and *Asclepiadoideae* genomes. Thus, the absence of this enzyme, together with the lack of detection of secologanin nor strictosidine in *P. lamerei* leaf extracts (Fig. 2D) suggests that an evolutionary loss of MIA biosynthesis may have occurred in non-*Rauvolfioideae* species among *Apocynaceae*, similarly to what happened in *Rubiaceae* family [58,69]. This statement is reinforced by the lack of many other MIA biosynthetic genes such as SGD, geissoschizine oxidase (GO), stemmadenine-*O*-acetyltransferase (SAT) or precondylocarpine acetate synthase (PAS) which probably did not evolved in the absence of the precursor strictosidine (Supplementary Table S21). It is worth noting that no SAT-like genes could be found in *Gelsemiaceae* nor *Rubiaceae* species as well as no GO-like genes could be found in *Rubiaceae* species. Such orthologous pattern suggests that enzymes modifying the strictosidine aglycone derivatives may have appeared independently in these families leading to family-specific MIA skeletons (*e.g.* quinoline-type in *Rubiaceae* or corynanthe-type in *Apocynaceae*).

### 2.3 Evolutionary divergence of *P. lamerei*

To gain a more comprehensive understanding of the evolution of specialized metabolites biosynthesis in *Apocynaceae*, a maximum-likelihood phylogenetic tree was established (Fig. 3A). Therefore, protein coding genes from 8 *Apocynaceae* species (3 *Rauvolfioideae*, 2 *Apocynoideae* and 3 *Asclepiadoideae*), 4 non-*Apocynaceae* MIA-producing species (2 *Rubiaceae*, 1 *Gelsemiaceae* and 1 *Nyssaceae*) and 4 non-*Apocynaceae* non-MIA producing species (*A. thaliana*, *C. canephora*, *S. lycopersicum* and *V. vinifera*) were compared. 24,413 orthogroups were defined from these 16 protein datasets covering 94.1% of all proteins. 387 of them were single-copy orthogroups and used to set up the phylogenetic tree. The resulting phylogenetic tree is in agreement with *Apocynaceae* classification based on other approaches including morphological characterization and plastome analysis (Fig. 3A) [3,70]. In addition to their common genomic features, *V. thouarsii* and *P. lamerei* are the first species to outgroup in *Rauvolfioideae* and non-*Rauvolfioideae* subfamilies.

The evolution of orthogroups was then analyzed along the phylogenetic tree (Fig. 3B–Supplementary Table S23). Orthogroups associated with shikimate and MEP pathways are present in all studied genomes suggesting that these pathways share a common evolutionary origin. Conversely, iridoid pathway-associated orthogroups are only identified in *Nyssaceae*, *Rubiaceae*, *Gelsemiaceae* and *Rauvolfioideae* species. Such distribution suggests that iridoid biosynthesis has emerged in the *Magnoliopsida* common ancestor and subsequently experienced independent losses in *Solanaceae* and non-*Rauvolfioideae* for instance. Obviously, orthogroups associated with MIA biosynthesis are mainly identified in *Rauvolfioideae* species. In addition, most of these MIA biosynthesis-associated orthogroups could also be found in *G. sempervirens*, *O. pumila* and *M. speciosa*. Interestingly, as the phylogenetic distance from *C. roseus* increases, fewer orthogroups associated with MIA biosynthesis are identified. This detection pattern therefore suggests an independent acquisition of post-strictosidine aglycone modifying enzymes, probably linked to the increase in gene contents in *Rauvolfioideae* species. This hypothesis could be strengthened by the future identification of genes coding for enzymes involved in MIAs biosynthesis in *Rubiaceae* and/or *Gelsemiaceae*, provided that the new orthogroups associated with these genes are not detected in *Rauvolfioideae* species.

### 2.4 Evolutionary scenario of biosynthetic gene clusters in *Apocynaceae*

To study the conservation of gene organization between chromosomes and species, a synteny analysis between *P. lamerei*, its close relative *V. thouarsii* [62] and *C. roseus* chromosome-scale genome has been performed [55] (Supplementary Figure S3). The dot plot comparing those species indicated an apparent collinearity between scaffolds. There was a grater collinearity between *P. lamerei* and *V. thouarsii* genomes than between *P. lamereii* and *C. roseus* genomes. While *P. lamerei* genome is only partially homologous with *C. roseus* genome, all *P. lamerei* scaffolds are homologous to *V. thouarsii* scaffolds despite some major insertion/deletion events. Hence, given their greater collinearity, their phylogenomic linkage, their similar genome size, their proportion of TE and LTR family ratio, *P. lamerei* and *V. thouarsii* are so far the most closely related species and therefore the closest species to the last common ancestor of *Rauvolfioideae* and non-*Rauvolfioideae.*

Given the low conservation of iridoid pathway genes and the absence of secologanin, we firstly examined the collinearity between *P. lamerei* and the genomic regions surrounding iridoid pathway genes in *C. roseus*. Although we were able to identify 3 highly conserved regions (Supplementary Figure S4), no syntenic region could be observed for 8HGO, IO, 7DLS, 7DLH, LAMT and SLS. It is worth noting that several tandem duplicates could be observed in both species for IRS and especially 7DLGT. These duplications could give rise to new functions via subfunctionalization throughout evolution [[71], [72], [73]]. It is noteworthy that despite a great conservation of G8H between *C. roseus* and *P. lamerei* (77.89%id, 100%cov), *P. lamerei* ortholog is greatly less expressed in all analyzed samples compared to *Rauvolfioideae* species (Supplementary Figure S5). Thus, the lack of collinearity in genomic regions surrounding most of iridoid pathway genes together with the low expression of G8H suggests a progressive degeneration of this pathway, the evolutionary processes of which remain to be discovered.

**Fig. 4:**
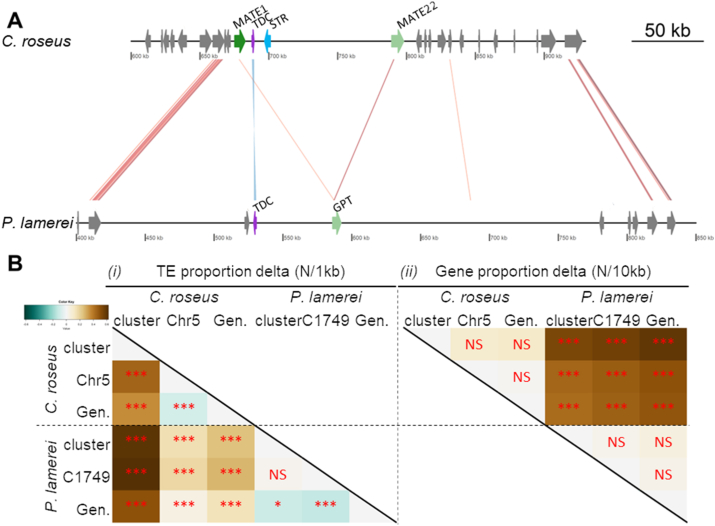
**Biosynthetic gene cluster evolution.** (A) Synteny between *C. roseus* and *P. lamerei* scaffolds that are involved in strictosidine biosynthesis. Blue: strictosidine synthase (STR), purple: tryptophan decarboxylase (TDC), green: MATE transporter, light green: transporter, grey: other function, GPT: glucose-6-phosphate transporter. (B) Transposable element (i, N/1 kb) and gene (ii, N/10 kb) proportion delta (Proportion in row subtracted to proportion in col) in the biosynthetic cluster, its associated scaffold/chromosome (Chr5: *C. roseus* chromosome 5, C1749: *P. lamerei* contig 1749) and the whole genome (Gen.). The proportion delta was calculated as follows: Δ = Proportion in the row condition-Proportion in the column condition. *P*-value: 0 “***” 0.001 “**” 0.01 “*” 0.05 “NS” 1. (For interpretation of the references to colour in this figure legend, the reader is referred to the Web version of this article.)

In fungi, bacteria and plants, genes involved in specific metabolic pathways frequently group together in the same genomic region [74]. These groups of genes, referred to as biosynthetic gene clusters, have been described in several MIA-producing plant species including the iconic *C. roseus* [52,53,55,56], *V. minor* [57], *R. stricta* [58] and *G. sempervirens* [53]. Microsyntenic study of these gene clusters enabled the identification of key enzymes in MIA biosynthesis as exemplified with the recent discovery of vincadifformine 16-hydroxylase in *V. minor* [57]. It is worth noting that genes encoding STR and TDC often cluster together as in *C. roseus*, *G. sempervirens*, *R. stricta* and *O. pumila* [52,53,55,56,58,69]. A recent single-cell approach in *C. roseus* unraveled a MATE transporter in this region that was found to be involved in secologanin transport from the cytosol to the vacuole [56]. By microsyntenic analysis between *C. roseus* and *P. lamerei*, we identified a similar [STR-TDC-MATE] region in *P. lamerei* contig 1749 (Fig. 4A). This chromosomal portion of *P. lamerei* is flanked by two highly conserved regions and, although it lacks any STR-like sequences, exhibits high TDC (reverse strand) conservation. Interestingly, only one transporter-like gene can be found in *P. lamerei* whereas two MATE genes have been annotated in this region in *C. roseus*, similarly to *G. sempervirens* region [53]. Overall, these preliminary observations thus strengthen the hypothesis that the absence of MIA production in *P. lamerei* and non-*Rauvolfioideae* species results from STR deletion through evolution in *Apocynaceae*.

In such a scenario, transposable elements might have played a major role in the proposed evolutionary mechanism. Indeed, while we observe a significantly higher proportion of genes in the *C. roseus* cluster, the syntenic region in *P. lamerei* exhibits a specific TE enrichment in the corresponding chromosome portion (Fig. 4B). Similar observations can be made throughout the genome which can explain the huge differences in genome size between these two species. It has to be noted that no significant differences in gene proportion in the cluster, its associated contig or chromosome and the whole genome can be observed for each species. Conversely, *P. lamerei* genome is significantly richer in TE than the cluster and contig 1749 and *C. roseus* cluster is significantly enriched in TE compared to chromosome 5 and its whole genome (Fig. 4B). This observation thus reinforces the putative implication of TE in [STR-TDC-MATE] biosynthetic cluster evolution. In addition, STR in *C. roseus* is flanked by two S0:0000544 helitrons (Supplementary Figure S6). Interestingly, the proportion of this autonomous rolling-circle DNA transposon is significantly higher in the *P. lamerei* region than the *C. roseus* region (*P. lamerei*: 0.38/kb, *C. roseus*: 0.08/kb, *P*-value: 3.005e-06) while being smaller. Helitrons exhibit ‘copy-and-paste’ and ‘cut-and-paste’ modes of transposition [75] as well as an excisive mode [76]. Hence, S0:0000544 helitron might have been a key player in the evolutionary mechanism leading to STR deletion in non-*Rauvolfioideae* species.

## 3 Conclusion

The comprehensive genomic exploration of *P. lamerei* genome in comparison with other *Apocynaceae* species offers intriguing insights into the evolutionary dynamics within this plant family. The assembly of the *P. lamerei* genome reveals a remarkable genome size of 968.6 Mb, making it the second largest *Apocynaceae* genome known to date. Interestingly, *P. lamerei* and the other non-*Rauvolfioideae* genomes challenge the norm for MIA-producing *Apocynaceae* genomes, presenting a lower-than-expected gene count such as the 16,176 protein-encoding genes for *P. lamerei*. This deviation from the typical gene count observed in MIA-producing *Apocynaceae* genomes raises questions about the genomic landscape of *P. lamerei* and its implications for specialized metabolism. The phylogenetic analysis places *P. lamerei* as an important species regarding the study of *Apocynaceae* evolution. Comparative genomics further reveals a similarity between *P. lamerei* and *V. thouarsii*, indicating a close relatedness among *Apocynaceae* species. The notable presence of TEs, especially LTR elements, in the *P. lamerei* (72.48%) and *V. thouarsii* (75.16% [62]) genomes raises questions about their role in shaping genomic architecture. It is likely that the common ancestor of these two species may have undergone horizontal transfer of TE, known to contribute to genome diversification [77]. This phenomenon could then have led to a sharp increase in genome size, which over time contracted through the removal of DNA in other *Apocynaceae* species such as *C. roseus*. As the insertion and removal of TE is often imprecise, it can indirectly affect the surrounding sequences which can lead to high duplication and reshuffling. These genomic rearrangements could explain the absence of certain MIA biosynthetic genes, including STR, in non-*Rauvolfioideae* species. Furthermore, TEs have been described to represent an important mechanism for gene evolution in rice and *Poaceae* [78,79]. Thus, they could also be a determinant factor in the emergence post-strictosidine aglycone modifying enzymes, as exemplified in rice [79]. This reinforces the potential interest of this genomic data set for the study of genetics and epigenetics mechanisms associated with the evolution, diversification and emergence of specialized metabolites biosynthetic pathways. Moreover, beyond widening our knowledge on MIA biosynthesis evolution, this new genome could improve our understanding of cardenolides emergence in *Asclepiadoideae* when this biosynthesis pathway is more elucidated.

## 4 Materials and methods

### 4.1 Sample collection, DNA extraction and sequencing

*Pachypodium lamerei* plants were purchased from *A l'ombre des figuiers* (achat-vente-palmiers.com). Nuclei were extracted from young leaves using a nuclei isolation protocol [80]. The nuclear pellet was resuspended in Qiagen buffer G2 with RNaseA and proteinaseK, and DNA extraction was further continued according to the instructions of Qiagen's Genomic Tip/100G protocol (Qiagen, Venlo, The Netherlands). DNAseq library was performed by Future Genomics Technologies (Leiden, The Netherlands) using Nextera Flex kit (Illumina, San Diego, USA) for Illumina sequencing and ONT 1D ligation sequencing kit (Oxford Nanopore Technologies Ltd, Oxford, United-Kingdom) for Nanopore sequencing. Illumina libraries were sequenced in pared-end mode (2x150 bp) using Illumina NovaSeq 6000 technology. ONT libraries were sequenced on Nanopore PromethION flowcell (Oxford Nanopore Technologies Ltd, Oxford, United-Kingdom) with the guppy version 3.0.3 high-accuracy basecaller.

### 4.2 *De novo* genome assembly

The *P. lamerei* genome assembly was performed by Future Genomics Technologies (Leiden, The Netherlands). After adapter removal using porechop [81], ONT reads were assembled using Flye assembler (v.2.8.3, [82]). Contig were twice polished with Illumina reads using pilon (v.1.23, [83]).

### 4.3 RNA extraction and sequencing

*Pachyopdium lamerei* plants were obtained from *ikhebeencactus* (ikhebeencactus.nl). RNA was extracted from liquid nitrogen flash-frozen leaves, spikes and roots using PureLink RNA mini kit (Thermo Fisher Scientific, Illkirch-Graffenstaden, France). RNA library construction and sequencing was performed using Illumina NovaSeq 6000 technology. Raw RNA-seq data have been deposited under the SRA accession numbers SRR25397418 (leaves), SRR25397417 (spikes), SRR25397419 (roots).

### 4.4 Gene model prediction and functional annotation

RNAseq reads were aligned to the *P. lamerei* reference genome using HiSAT2 (2.2.1 [84]). Subsequently, transcriptome was assembled for each alignment using StringTie (v2.1.7 [85]) and merged using stringtie merge option. A combination of BLASTX on predicted transcripts and BLASTp on TransDecoder (v5.5.0 [86]) predicted ORFs against the Uniprot database as well as hmmscan (v3.1b2 [87]) against the PFAM database (https://pfam.xfam.org/) was used to assess putative function of each gene model.

A similar strategy was used for gene modeling and gene functional annotation of *A. venetum*, *A. syriaca* and *C. procera*. Genomic sequence for each species was retrieved from GenBank accession numbers GCA_019593545.1, GCA_027405835.1 and GCA_004801955.1, respectively. RNAseq reads were retrieve for each species from SRA accession numbers: SRR17163778 (leaves), SRR17163779 (stems) and SRR17163780 (roots) for *A. venetum*; SRR5117431 (buds) for *A. syriaca*; and SRR8281638, SRR8281639, SRR8281640, SRR8281641, SRR8281642 and SRR8281643 (all corresponding to leaves under different salinity conditions) for *C. procera*.

### 4.5 Assembly completeness assessment

Merqury (v. 1.3 [88]) and the stat program from BBMap tool (v.38.94 [89]) were used to assess *P. lamerei* genome assembly quality. To evaluate assembly and gene model completeness, Benchmarking Universal Single-Copy Orthologs (BUSCO v.5.2.2 [90]) with default parameters was used using a plant-specific database of 2326 single copy orthologs (eudicots_odb10). Gene model statistics were retrieved using agat_sp_statistics from the AGAT package (v.0.8.0 [91]).

### 4.6 Transposable elements prediction and annotation

Transposable elements (TE) were identified and annotated using the sensitive mode of Extensive *de novo* TE annotator (EDTA v.1.9.5 [92]). This pipeline combines long-terminal repeat (LTR) annotation using LTR_finder (v. 1.07 [93]) and LTRharvest included in GenomeTools (v.1.5.10 [94]); terminal inverted repeat annotation (TIR) using Generic repeat finder (v.1.0 [95]) and TIR-learner (v.2.5 [96]); and Helitrons annotation using HelitronScanner (v.1.1 [97]). Apart from that, TE size thresholds are employed to avoid erroneous findings. Tandem repeats and short sequences are thereby defined as TIR shorter than 80 bp, LTR shorter than 100 bp and helitrons shorter than 100 bp. LTR are further filtered using LTR_retriever (v.2.9.0 [98]) to prevent erroneous LTR findings. If TIR candidates do not exceed 600 bp, they are categorized as MITEs. EDTA advanced filters are used for additional filtering TIR and Helitrons (for further information, see Ref. [92]). The TE library that was obtained is then used to mask the genome. RepeatModeler (v.2.0.1, default settings [99]) is then used on the unmasked portion of the genome to find non-LTR retrotransposons and unclassified TE that have eluded structure-based TE identification methods. Finally, gene-related sequences are removed by EDTA using the supplied CDS sequences.

### 4.7 Whole-genome duplication analysis

Transcript sequences of *P. lamerei*, *A. venetum*, *A. syriaca*, *C. procera*, *V. thouarsii* [62], *V. minor* [57], *C. roseus* [55], *Arabidopsis thaliana* [100], *Mytragyna speciosa* [101], *Solanum lycopersicum* [102], *C. acuminata* [103], *C. gigantea* [104], *G. sempervirens* [53], *Vitis vinifera* [105] and *Ophiorrhiza pumila* [69] were used to assume whole genome duplication (WGD) events using the DupPipe pipeline [106]. Each dataset was subject to discontiguous MegaBLAST [107,108] to find duplicated gene pairs (40% sequence similarity over 300bp). Using BLASTx (v.2.6.0–1 [109]), the open reading frame of each gene pair was determined from the NCBI's plant RefSeq protein database (as of May 21, 2021), preserving solely the best hit sequence (sequence similarity threshold: 30% across 150 aa). Following that, DNA sequence alignment was done using GeneWise [110] against the best hit homologous protein and its translation. For each gene pair, MUSCLE (v.3.6 [111]) aligned the amino acid sequences, which then served as a guide for RevTrans (v.1.4 [112]) to align the nucleic acids. In order to assess the divergence times between gene pairs, substitutions per synonymous site (Ks) using the Codeml's F3x4 model from the PAML software (v.4.9 [113]) were calculated.

### 4.8 Orthology analysis and phylogenetic tree reconstruction

Protein sequences of at least 30 amino acids from *P. lamerei*, *A. venetum*, *A. syriaca*, *C. procera*, *V. thouarsii* [62], *V. minor* [57], *C. roseus* [55], *A. thaliana* [100], *M. speciosa* [101], *S. lycopersicum* [102], *C. acuminata* [103], *C. gigantea* [104], *G. sempervirens* [53], *V. vinifera* [105] and *O. pumila* [69] were compared in order to build gene families. CD-HIT (v.4.7 [114]) was used for each species. In each CD-HIT cluster, the longest representative protein was selected. The subsequent protein datasets were used as input for OrthoFinder (v.2.5.4 [115]) using the following parameters: S diamond -M msa -A muscle -T raxml-ng. From the 387 single-copy orthogroups, a maximum-likelihood phylogenetic tree was built. In order to identify orthogroup loss and growth across the phylogenetic tree, Cafe5 (v.4.2.1 [116]) was employed.

### 4.9 Synteny analysis

*P. lamerei* genome was aligned with *V. thouarsii* and *C. roseus* genome using minimap2 (v.2.24 [117]) and the following options: cx asm20 --cs. D-Genies (https://dgenies.toulouse.inra.fr/ [118]) was used to visualize the generated paf file, selecting hits with at least 80% identity and ranking contigs by size. Syntenic regions between STR/TDC/MATE1 gene cluster for *C. roseus* and TDC/MATE1 gene cluster for *P. lamerei* were compared using BLASTN (v.2.6.0–1 [109]). The resulting hits between the clusters were filtered to only include alignments with an e-value of 1e-6 and alignment length of 1500 bp and alignments were visualized using the R genoPlotR library (v.0.8.11 [119]). Gene and TE in microsyntenic regions was performed by comparing their proportion in the region of interest to their proportion in the corresponding contig by an exact Poisson test with the poisson.test function implemented in stat package (v. 4.1.1) in R (v. 4.1.1 [120]).

### 4.10 Transcript Abundance estimation

Salmon (v.0.6.0 [121]) with -biasCorrect and -vbo options was used to count RNAseq reads after they were pseudo-aligned onto the predicted transcripts. Abundance estimates were established as transcripts per million (TPM) and are presented in Supplementary Table S24.

### 4.11 Targeted metabolomics analysis

To attest the presence of MIA and cardenolides, a targeted metabolomic analysis was carried out. Metabolites were extracted by vortexing 50 mg of lyophilized leaf powder with 1 mL of 0.1% formic acid methanol solution. Extracts were injected into the ultra-performance liquid chromatography system ACQUITY (Waters, Milford, MA, USA) coupled to the single quadrupole mass spectrometer SQD2 (Waters, Milford, MA, USA) using conditions described in Ref. [122] over a 18 min gradient. The presence of tryptophan, secologanin and strictosidine, three key metabolites in MIA biosynthesis, as well as progesterone for cardenolides biosynthesis, was then checked by injecting the corresponding standards and a selected ion monitoring mode was used to collect data for tryptophan (*m*/*z* 205), secologanin (*m*/*z* 389), strictosidine (*m*/*z* 531.2) and progesterone (*m*/*z* 315).

To attest the presence of flavonoids, similar analysis was carried out on *C. roseus* and *P. lamerei*. Young and old leaves of *P. lamerei* and *C. roseus* and *C. roseus* flowers were collected and immediately flash-frozen in liquid nitrogen. Samples were freeze-dried for 48 h and subsequently ground using MM400 ball grinder (Retsch GmbH, Haan, Germany). 20 mg of powder for each sample were then added to 1 mL of 80% methanol and sonicated for 30 min. After 10 min centrifugation at 18,000×*g* and 4 C°, 600 μL of supernatant were retrieved and five-fold diluted with the mobile phase (milliQ water – acetonitrile 5% - formic acid 0.1%). Extracts were then injected into the ultra-performance liquid chromatography system ACQUITY (Waters, Milford, MA, USA) coupled to the single quadrupole mass spectrometer XEVO (Waters, Milford, MA, USA) using conditions described in Ref. [123]. A selected ion monitoring mode was used to collect data for phenylalanine (*m*/*z* 166), naringenin (*m*/*z* 273), kaempferol (*m*/*z* 285), kaempferol-3-glucoside (*m*/*z* 449), quercetin (*m*/*z* 301), quercetin-3-glucoside (*m*/*z* 465), pelargonidin (*m*/*z* 272), cyanidin (*m*/*z* 288), cyanidin-3-glucoside (*m*/*z* 485), catechin (*m*/*z* 291) and epicatechin (*m*/*z* 291). To confirm metabolite identification, the corresponding standards were injected for all targets.

## Grant information

This work was supported by EU 10.13039/501100007601Horizon 2020 research and innovation program [MIAMi project-Grant agreement N°814645]; 10.13039/501100012495ARD CVL Biopharmaceutical program of the Région Centre-Val de Loire [ETOPOCentre project]; ARP-IR from the Région Centre-Val de Loire [ScaleBio project] and ANR [project MIACYC – ANR-20-CE43-0010].

## Data availability statement

Raw DNA-seq data, raw RNA-seq data and the genome assembly have been deposited in the NCBI database under the BioProject accession number: PRJNA997810 (https://www.ncbi.nlm.nih.gov/bioproject/PRJNA997810). The genome annotation, its functional annotation, transcripts sequences, predicted CDS and protein sequences as well as database containing functionally characterized proteins involved in shikimate, methylerythritol phosphate (MEP), iridoid, MIA, steroids and cardenolide pathways are available on the figshare (Cuello et al., 2023, https://doi.org/10.6084/m9.figshare.23126816).

## CRediT authorship contribution statement

**Clément Cuello:** Writing – review & editing, Writing – original draft, Visualization, Methodology, Investigation, Formal analysis, Data curation. **Hans J. Jansen:** Methodology, Investigation, Formal analysis, Data curation. **Cécile Abdallah:** Writing – review & editing, Methodology, Investigation. **Duchesse-Lacours Zamar Mbadinga:** Writing – review & editing, Investigation. **Caroline Birer-Williams:** Writing – review & editing, Investigation. **Mickael Durand:** Writing – review & editing, Investigation. **Audrey Oudin:** Investigation. **Nicolas Papon:** Writing – review & editing, Investigation. **Nathalie Giglioli-Guivarc'H:** Writing – review & editing, Investigation. **Ron P. Dirks:** Validation, Formal analysis, Data curation. **Michael Krogh Jensen:** Project administration, Funding acquisition, Conceptualization. **Sarah Ellen O'Connor:** Project administration, Funding acquisition, Conceptualization. **Sébastien Besseau:** Writing – review & editing, Validation, Supervision, Investigation, Conceptualization. **Vincent Courdavault:** Writing – review & editing, Writing – original draft, Validation, Supervision, Project administration, Funding acquisition, Conceptualization.

## Declaration of competing interest

The authors declare the following financial interests/personal relationships which may be considered as potential competing interests:Vincent Courdavault reports financial support was provided by Horizon Europe. Michael Krogh Jensen reports a relationship with BioMIA that includes: board membership and employment. Ron Dirks reports a relationship with Future Genomics Technologies that includes: board membership and employment. Hans Jensen reports a relationship with Future Genomics Technologies that includes: board membership and employment. If there are other authors, they declare that they have no known competing financial interests or personal relationships that could have appeared to influence the work reported in this paper.
