# Supplementary material for: The Madagascar palm genome provides new insights on the evolution of Apocynaceae specialized metabolism
Source: Heliyon. 2024 Mar 14;10(6):e28078. doi: 10.1016/j.heliyon.2024.e28078 (PMC10963385; doi:10.1016/j.heliyon.2024.e28078)
Supplement: Multimedia component 1 [file mmc1.pptx]

## Slide 1
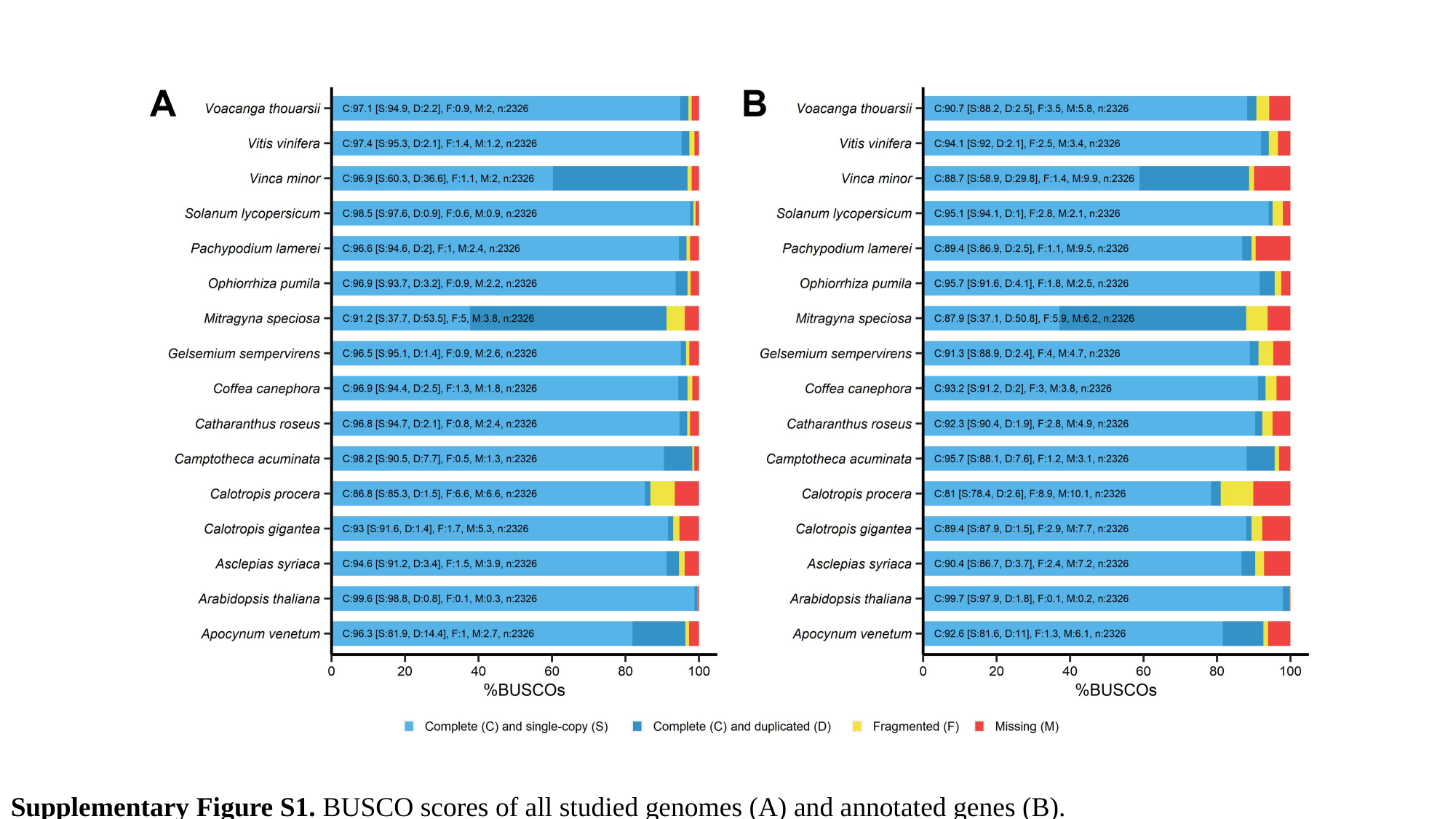

Supplementary Figure S1. BUSCO scores of all studied genomes (A) and annotated genes (B).

## Slide 2
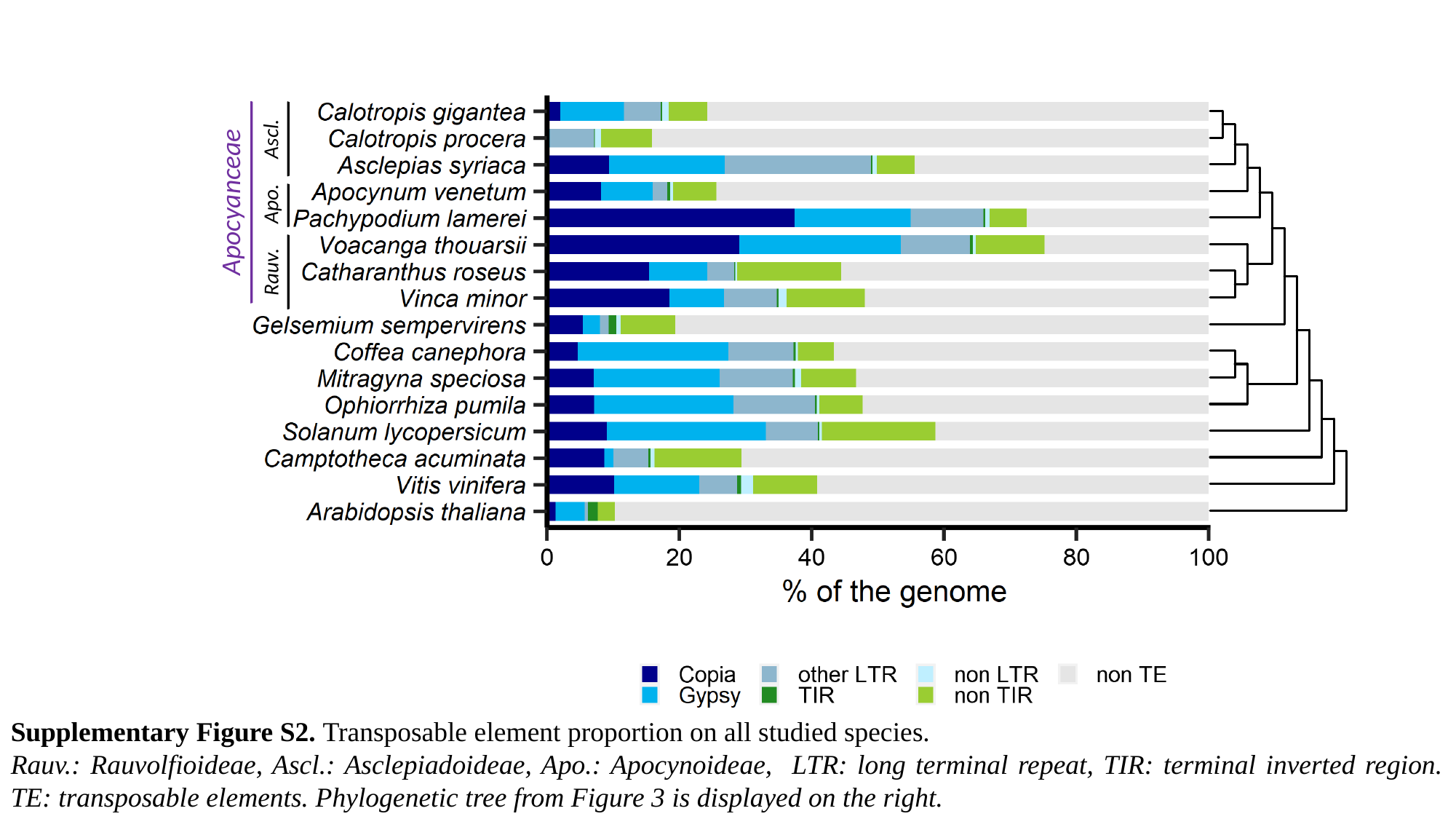

Ascl.
Apocyanceae
Apo.
Rauv.
Supplementary Figure S2. Transposable element proportion on all studied species.
Rauv.: Rauvolfioideae, Ascl.: Asclepiadoideae, Apo.: Apocynoideae, LTR: long terminal repeat, TIR: terminal inverted region. TE: transposable elements. Phylogenetic tree from Figure 3 is displayed on the right.

## Slide 3
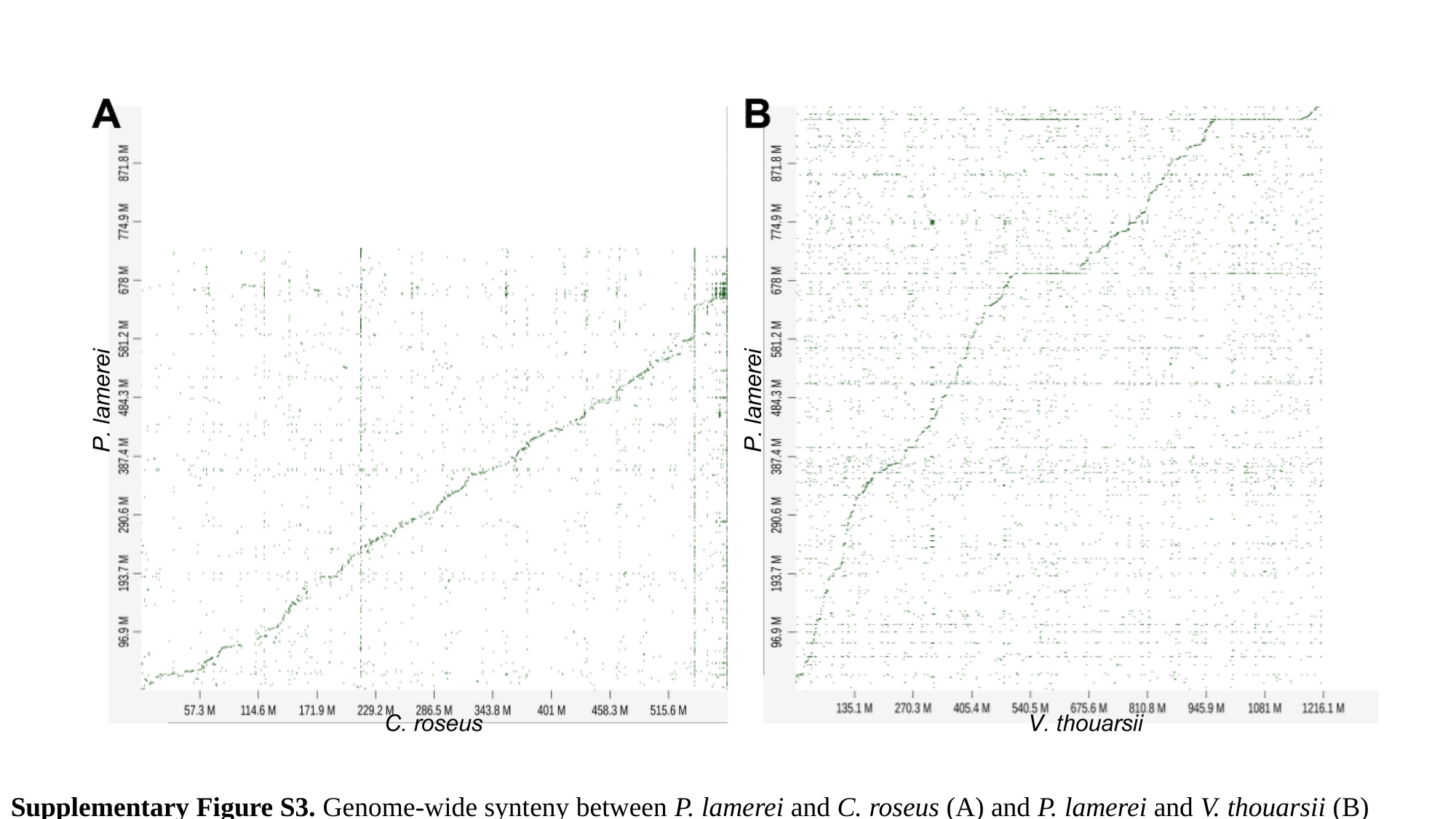

Supplementary Figure S3. Genome-wide synteny between P. lamerei and C. roseus (A) and P. lamerei and V. thouarsii (B)

## Slide 4
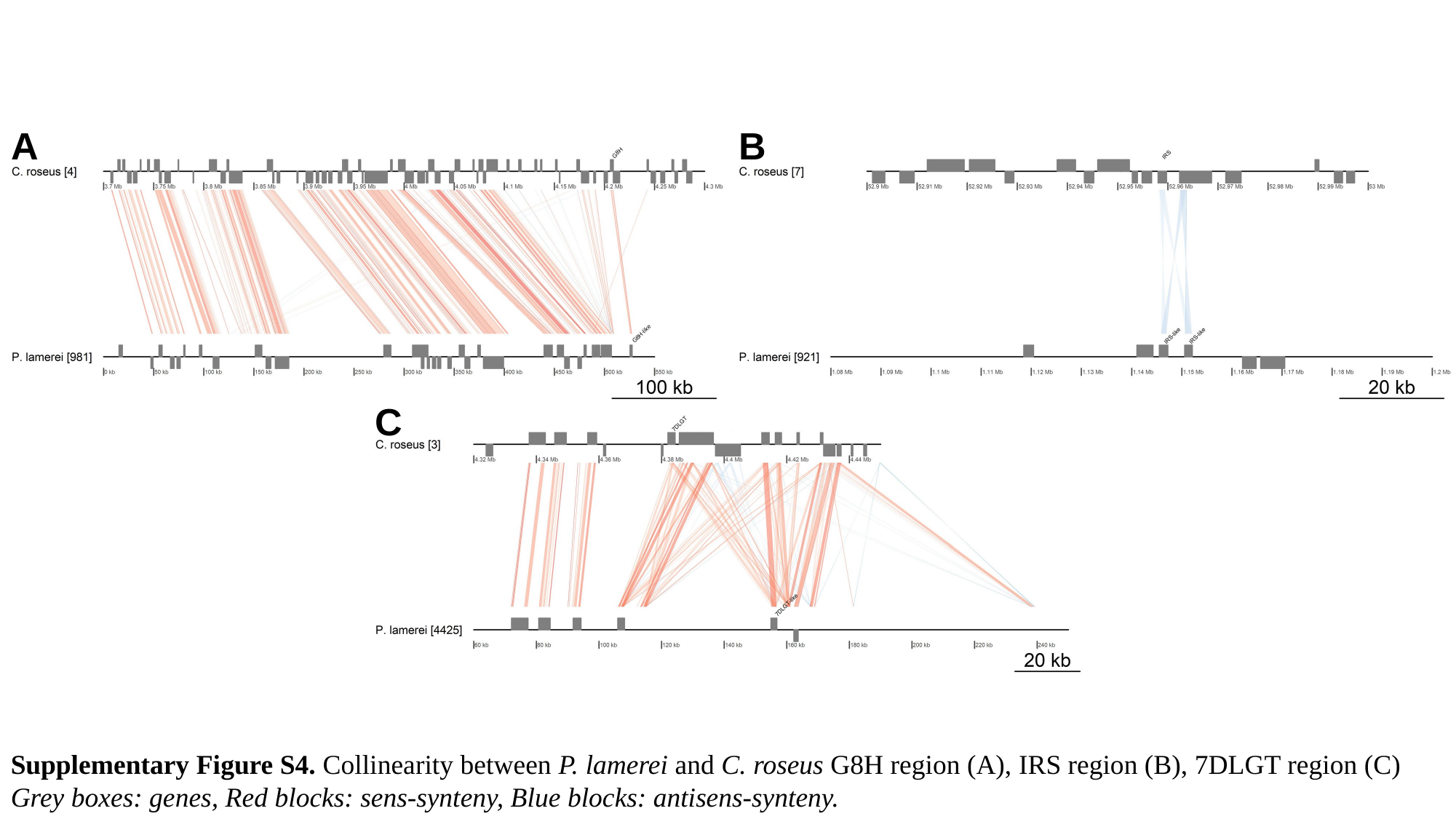

A
B
G8H
G8H-like
C
Supplementary Figure S4. Collinearity between P. lamerei and C. roseus G8H region (A), IRS region (B), 7DLGT region (C)
Grey boxes: genes, Red blocks: sens-synteny, Blue blocks: antisens-synteny.

## Slide 5
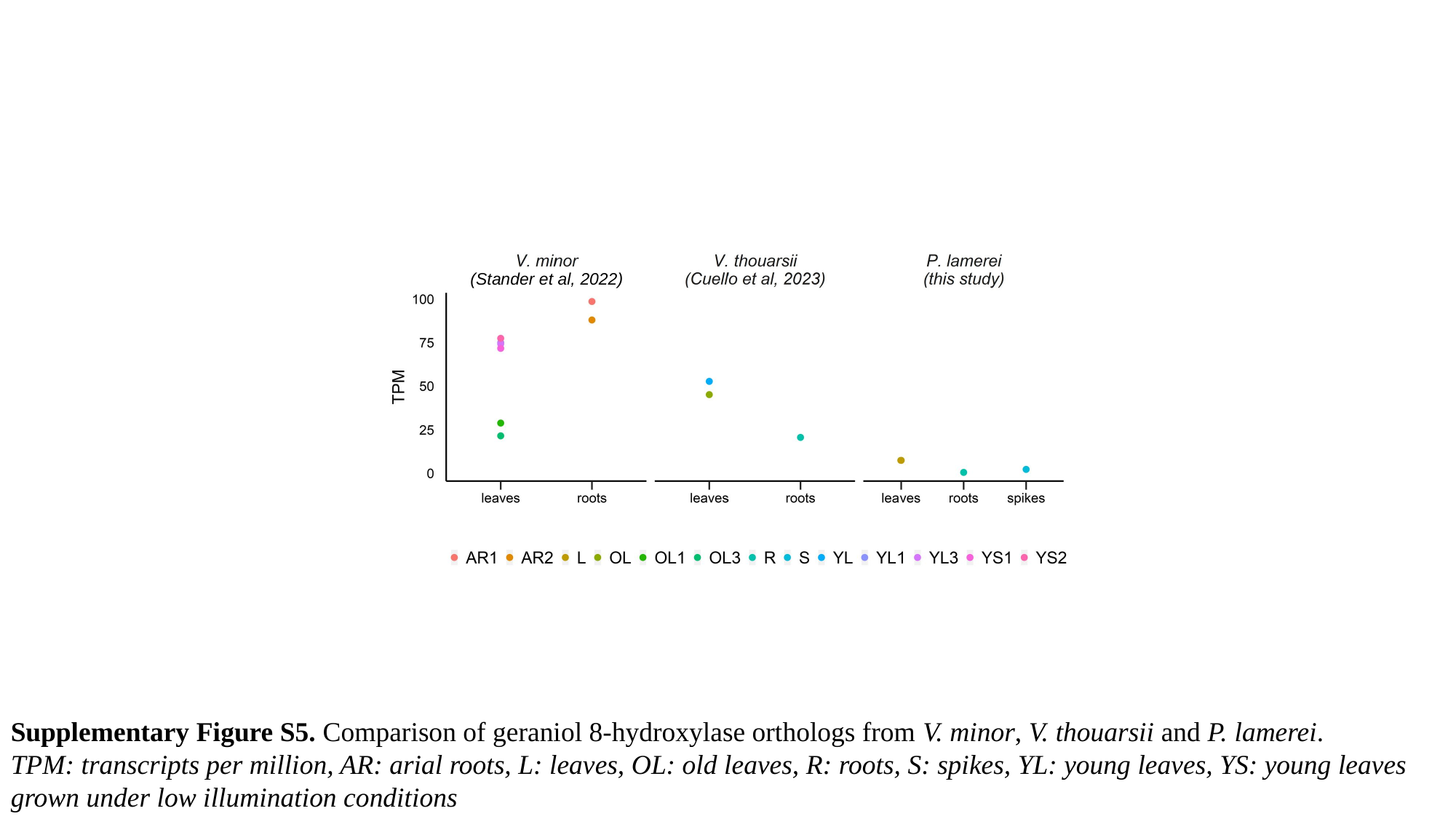

(Stander et al, 2022)
Supplementary Figure S5. Comparison of geraniol 8-hydroxylase orthologs from V. minor, V. thouarsii and P. lamerei.
TPM: transcripts per million, AR: arial roots, L: leaves, OL: old leaves, R: roots, S: spikes, YL: young leaves, YS: young leaves grown under low illumination conditions

## Slide 6
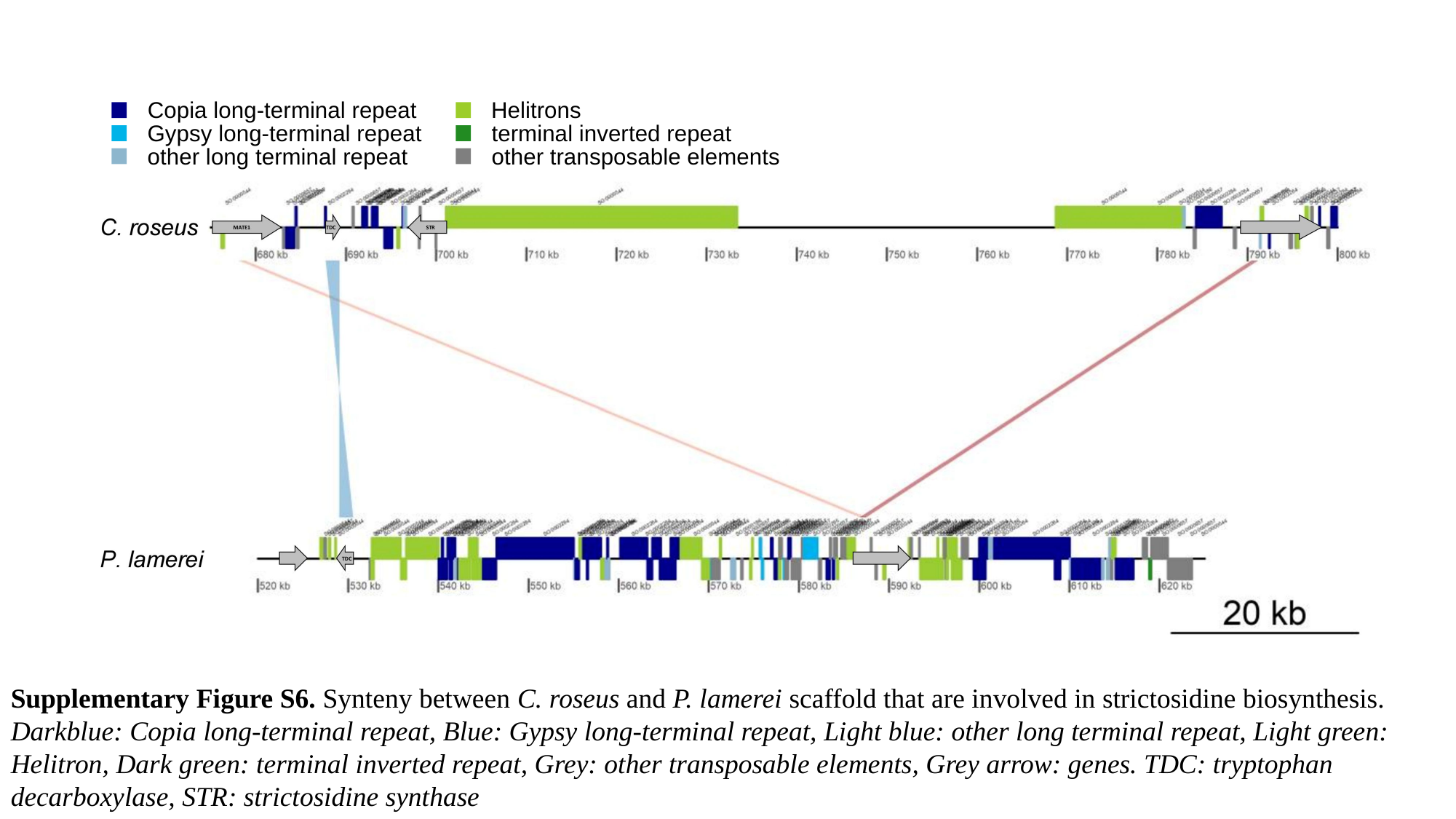

Copia long-terminal repeat
Helitrons
Gypsy long-terminal repeat
terminal inverted repeat
other long terminal repeat
other transposable elements
Supplementary Figure S6. Synteny between C. roseus and P. lamerei scaffold that are involved in strictosidine biosynthesis. Darkblue: Copia long-terminal repeat, Blue: Gypsy long-terminal repeat, Light blue: other long terminal repeat, Light green: Helitron, Dark green: terminal inverted repeat, Grey: other transposable elements, Grey arrow: genes. TDC: tryptophan decarboxylase, STR: strictosidine synthase
